# Structural racism is associated with adverse postnatal outcomes among Black preterm infants

**DOI:** 10.1038/s41390-022-02445-6

**Published:** 2022-12-28

**Authors:** Kayla L. Karvonen, Safyer McKenzie-Sampson, Rebecca J. Baer, Laura Jelliffe-Pawlowski, Elizabeth E. Rogers, Matthew S. Pantell, Brittany D. Chambers

**Affiliations:** 1 Department of Pediatrics, University of California San Francisco, San Francisco, CA USA; 2 California Preterm Birth Initiative, San Francisco, CA USA; 3 Department of Epidemiology and Statistics, University of California San Francisco, San Francisco, CA USA; 4 Department of Pediatrics, University of California San Diego, La Jolla, CA USA; 5 Department of Human Ecology, University of California Davis, Davis, CA USA

## Abstract

**Background:**

Structural racism contributes to racial disparities in adverse perinatal outcomes. We sought to determine if structural racism is associated with adverse outcomes among Black preterm infants postnatally.

**Methods:**

Observational cohort study of 13,321 Black birthing people who delivered preterm (gestational age 22–36 weeks) in California in 2011–2017 using a statewide birth cohort database and the American Community Survey. Racial and income segregation was quantified by the Index of Concentration at the Extremes (ICE) scores. Multivariable generalized estimating equations regression models were fit to test the association between ICE scores and adverse postnatal outcomes: frequent acute care visits, readmissions, and pre- and post-discharge death, adjusting for infant and birthing person characteristics and social factors.

**Results:**

Black birthing people who delivered preterm in the least privileged ICE tertiles were more likely to have infants who experienced frequent acute care visits (crude risk ratio [cRR] 1.3 95% CI 1.2–1.4), readmissions (cRR 1.1 95% CI 1.0–1.2), and post-discharge death (cRR 1.9 95% CI 1.2–3.1) in their first year compared to those in the privileged tertile. Results did not differ significantly after adjusting for infant or birthing person characteristics.

**Conclusion:**

Structural racism contributes to adverse outcomes for Black preterm infants after hospital discharge.

**Impact statement:**

- Structural racism, measured by racial and income segregation, was associated with adverse postnatal outcomes among Black preterm infants including frequent acute care visits, rehospitalizations, and death after hospital discharge.
- This study extends our understanding of the impact of structural racism on the health of Black preterm infants beyond the perinatal period and provides reinforcement to the concept of structural racism contributing to racial disparities in poor postnatal outcomes for preterm infants.
- Identifying structural racism as a primary cause of racial disparities in the postnatal period is necessary to prioritize and implement appropriate structural interventions to improve outcomes.

## Introduction

Racial disparities in adverse perinatal and postnatal outcomes including prematurity, low birthweight, preterm comorbidities, infant mortality, and health care utilization have been previously described.^1–5^ Racial disparities often persist despite adjusting for birthing person characteristics, including medical co-morbidities and socioeconomic factors.^2,4,5^ Although genetic etiologies of racial disparities in perinatal outcomes were historically entertained, it is now understood that race is a socio-political construct without a genetic basis.^6,7^ Once a historically under-recognized concept, racism in all its forms, structural, interpersonal, and internal, is now recognized as a core social determinant of health (SDH) with substantial health consequences for pediatric populations.^8,9^ Structural racism is defined as systematically discriminatory laws and practices that have resulted in a disparate distribution of goods, services, and opportunities for racial groups.^10^ Structural racism has been directly associated with poor perinatal outcomes including preterm birth (PTB), infant mortality, and small for gestational age (SGA) infants.^11–17^

Structural racism has been quantified by measuring distribution of resources and opportunities such as employment, education, income, and incarceration.^11,18–20^ One such measure of structural racism is the Index of Concentration at the Extremes (ICE), a metric developed by Massey et al. and further modified by Krieger et al., that measures spatial social polarization by quantifying extremes of privilege among social groups using race and income data (ICE race + income).^20,21^ In previous perinatal literature, ICE operationalized as a proxy of structural racism has been associated with preterm birth, infant mortality, and a combined outcome of neonatal mortality and severe preterm comorbidity.^15,22–24^ Less is known about how structural racism impacts preterm infants after their initial hospitalization.

Previously, we described that Black preterm infants were at higher risk of frequent acute care visits, readmissions, and death after hospital discharge in their first year of life when compared to white preterm infants.^4,5^ Despite adjusting for several medical and social covariates, our findings persisted, and we hypothesized that structural racism was a root cause of the racial and ethnic disparities observed. Although structural racism is associated with adverse perinatal outcomes like preterm birth and infant mortality for Black infants, less is known about how structural racism may continue to contribute to the health and wellbeing of Black preterm infants after hospital discharge. Thus, in this study, we investigate if structural racism, measured by ICE race + income, is associated with previously described adverse postnatal infant outcomes including frequent acute care visits, readmissions, and pre- and post-discharge mortality in the first year of life.

## Methods

### Study population

**Fig. 1:**
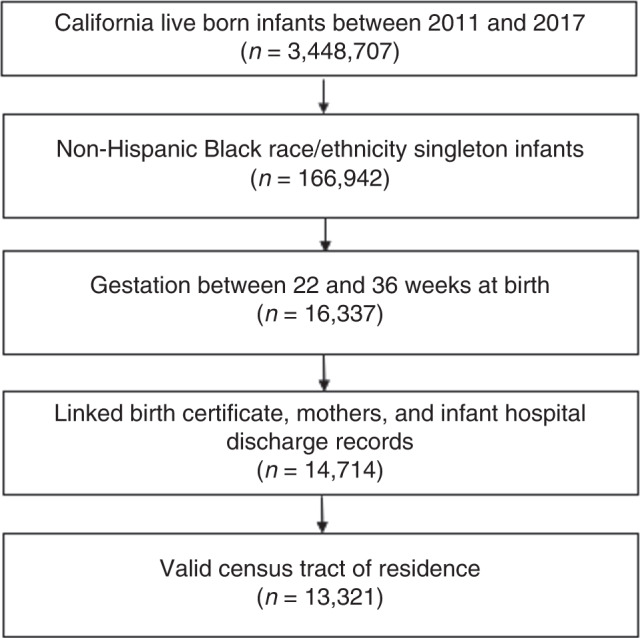
Sample selection. Flow chart of sample selection criteria.

The data for our cohort study was drawn from birthing persons, a term that recognizes not all birthing people identify as women, who delivered liveborn infants in California between 2011 and 2017 (*n* = 3,448,707) using a birth cohort database. The database maintained by the Office of Statewide Health Planning and Development includes birth certificate, infant hospital/emergency department, birthing person hospital/emergency department, and infant death records up to the infant’s first year of life. The sample was merged with census tract data available from the American Community Survey (ACS, 2011–2017) to generate ICE scores by census tract. The sample was restricted to live born singletons of non-Hispanic Black race/ethnicity birthing persons (*n* = 166,942) with gestational ages <37 weeks (*n* = 16,337), with records available for both infants and birthing person pairs (*n* = 13,321, Fig. 1). Non-Hispanic Black race/ethnicity birthing persons were prioritized in the study as this group has been subject to extreme structural racism in the U.S. and a high risk of adverse obstetric and neonatal outcomes. Data from ACS regarding non-Hispanic white birthing persons was also used to calculate ICE race + income scores, although this group was not included in the study population.

### Exposure and outcomes

Self-reported race and ethnicity was abstracted from the infant’s birth certificate. Race and ethnicity were organized into the following groups for our analysis: non-Hispanic white (which we will refer to as “white”) and non-Hispanic Black (which we will refer to as “Black”). The ICE metric measures Black-white disparities and thus Hispanic, Asian, American Indian and Alaska Natives, Native Hawaiian and Pacific Islander, and multiracial infants were excluded. Birthing person demographic characteristics were obtained from the birth cohort database. The primary outcomes included more than or equal to two acute care and/or emergency department (ED) visits based on a modified prior definition of frequent ED visits,^5,25^ hospital readmission, less than 7 day mortality, and pre- and post-discharge mortality. All outcomes occurred in the first year of life and were obtained from the birth cohort database.

### Covariates

Birthing person age was categorized into less than 18 years, 18–34 years, and greater than 34 years. Birthweight was used to assess in utero growth and was categorized into small for gestational age (SGA), average for gestational age (AGA), and large for gestational age (LGA) defined by less than 10th percentile, 10–90th percentile, greater than 90th percentile birth weights, respectively.^26^ Birthing person characteristics included body mass index (BMI), categorized as underweight (less than18.5 kg/m2), normal weight (18.5–24.9 kg/m2), overweight (25.0–29.9 kg/m2), and obese (30.0 kg/m2). Other covariates collected with ICD-9 and ICD-10 codes on hospital records for birthing people included any smoking, alcohol, or illicit drug use, chronic or gestational hypertension, chronic or gestational diabetes mellitus. Adequacy of prenatal care was a binary outcome defined by Kotelchuck et al.^27^ Social factors included: highest level of completed education (less than high school education, high school graduate, and more than high school education), insurance coverage for delivery (public, private, or other), and participation in the federal supplemental nutrition assistance program, Women, Infants, and Children (WIC).

### Index of concentration at the extremes (ICE)

ICE race + income scores were generated using race and income data for census tracts derived from The American Community Survey (2011–2017). ICE measures spatial social polarization by quantifying extremes of privilege among social groups in a single metric.^20,21^ The following formula is used to calculate ICE: ICE_i_ = (A_i_-P_i_/T_i_) where A_i_ represents the number of persons belonging to most privileged extreme, P_i_ is the number of persons who belong to the least privileged extreme in the _i_th census tract. T_i_ is the total population in the _i_th census tract.

This study uses the combined race and income ICE measure as proposed by Krieger et al.^21^ The most privileged race and income group was defined as non-Hispanic white individuals with annual income >$100,000 and the least privileged group was defined as non-Hispanic Black individuals with annual income of <$25,000 annually. Annual incomes of <$25,000 and >$100,000 represent the 20th and 80th percentiles of household income, respectively. ICE is a continuous variable that ranges from −1 to 1, where −1 represents least privileged and 1 is most privileged. ICE race + income scores were categorized into three tertiles based on sample distributions (*n* = 13,321) of these measures from tertile 1 (least privileged) to tertile 3 (most privileged). ICE scores are calculated and assigned to each census tract, thus ICE scores represent the degree of racial and income segregation and inequity in the area in which each birthing persons live.

### Statistical analysis

We computed summary statistics, including the mean, standard deviation, minimum and maximum for each ICE race + income tertile for the entire study sample, and for all California live births. In addition, we computed the proportion of covariates and outcomes under study. Moreover, we computed the percent of adverse postnatal outcomes by ICE income + race tertile and a chi-square test to examine differences among the tertiles.

We then fit four sequential generalized estimating equations regression models with robust standard errors and an exchangeable correlation structure to generate risk ratios (RR) and 95% confidence intervals (CI) testing the association between ICE race + income score for the census tract in which a birthing person lives and adverse postnatal outcomes. The first model was unadjusted, while the second was adjusted for infant sex, gestational age and growth. The third model further adjusted for birthing person age, BMI, smoking/drug or alcohol use, adequacy of prenatal care as well as chronic or gestational hypertension and diabetes. The fourth and final model was additionally adjusted for the highest level of birthing person education, insurance coverage for delivery, and WIC participation. All data were analyzed in Stata version 16.1 (StataCorp LP, College Station, Texas).

Methods and protocols for the study were approved by the Committee for the Protection of Human Subjects of the Health and Human Services Agency of the State of California as well as the Institutional Review Board of the University of California, San Francisco.

## Results

Among the 3,448,707 live born infants in California between 2011 and 2017, 16,337 infants were non-Hispanic Black singletons born between 22 and 36 weeks gestation, of those 13,321 had valid census tract and birth cohort data and thus were included in the study (Fig. 1 online). ICE race + income distributions for the entire California live birth population calculated for each census tract compared to the study sample are displayed in Table 1. In California, ICE race + income score distributions for each census tract ranged from −0.49 to +1, and similarly the ICE race + income score distributions for the census tracts in which the study sample of birthing persons lived ranged from −0.48 to +1.

**Table 1:** ICE distributions for California and study sample 2011–2017.

	California population (*n* = 3,167,807)				
	*n*	Mean	SD	Minimum	Maximum
ICE race + income					
Tertile 1	1,055,954	−0.03	0.06	−0.49	0.02
Tertile 2	1,055,959	0.08	0.04	0.02	0.15
Tertile 3	1,055,894	0.29	0.11	0.15	1.00

	Sample population (*n* = 13,321)				
	*n*	Mean	SD	Minimum	Maximum
ICE race + income					
Tertile 1	4441	−0.15	0.08	−0.48	−0.06
Tertile 2	4441	−0.01	0.03	−0.06	0.04
Tertile 3	4439	0.15	0.11	0.04	1.00

Demographic characteristics of Black birthing people and their infants are described in Table 2. In this sample, 19.4% of birthing people delivered very preterm (less than 32 weeks gestation) and 80.6% delivered late and moderately preterm (32–36 weeks gestation) infants. SGA infants were overrepresented at 14.4%, whereas LGA infants were underrepresented at 7% compared to their percentile definitions. Birthing people in this sample were mostly between 18–34 years (78.4%), overweight or obese (54.2%), did not smoke, use alcohol, or drugs during pregnancy (82%), had Medi-Cal insurance (59.7%), participated in WIC (65.8%), and had adequate prenatal care (70.4%). Similar proportions of birthing people had at least a high school education compared to less than a high school education (49.8% vs 47.2%), and had pre-existing or gestational diabetes (41.0% vs 59.0%).

**Table 2:** Black birthing people sample characteristics in 2011–2017.

Sample	*n*	%
Male infant sex	6827	51.3
GA		
<32 weeks	2584	19.4
32–36 weeks	10,737	80.6
<37 weeks	13,321	100.0
Birthweight^a^		
SGA	1919	14.4
AGA	10,470	78.6
LGA	932	7.0
Birthing person age		
<18 years	298	2.2
18–34 years	10,446	78.4
>34 years	2576	19.3
Birthing person education		
Less than or equal to 12 years	6292	47.2
More than 12 years	6637	49.8
Missing	392	2.9
Birthing person BMI		
Underweight	587	4.4
Normal weight	4666	35.0
Overweight	3052	22.9
Obese	4174	31.3
Missing	842	6.3
Any smoking, alcohol use or drug use during pregnancy		
Yes	2395	18.0
No	10,926	82.0
Insurance		
Medi-Cal	7951	59.7
Not Medi-Cal	4357	32.7
Missing	1013	7.6
WIC participation		
Yes	8760	65.8
No	4468	33.5
Unknown	93	0.7
Any hypertension or diabetes		
Yes	5459	41.0
No	7862	59.0
Prenatal Care^b^		
Adequate/adequate plus	9378	70.4
Intermediate/inadequate	3319	24.9
Missing	624	4.7
Infant outcomes		
<7d mortality	298	2.2
Pre-discharge mortality	408	3.1
>/=2 acute care visits	3546	26.6
Rehospitalization	2635	19.8
Post-discharge mortality	115	0.9

^a^Ref. 26

^b^Ref. 27

Considering the infant outcomes, death was a rare event, with few infants dying in the first 7 days of life (2.2%), before discharge (3.1%), and after discharge (0.86%). However, rehospitalization (19.8%) and acute care visits were more common (26.6%).

Black birthing people living in the least privileged ICE race + income tertiles consistently had the highest percentage of adverse birth outcomes (Table 3). The proportion of acute care visits, readmission, and mortality were significantly different by tertile, confirmed with a chi-squared test (*p* values < 0.001, 0.03, and 0.02, respectively). Whereas the percentage of adverse infant outcomes for less than 7 days and pre-discharge mortality did not statistically significantly vary by tertile.

**Table 3:** Percent of adverse birth outcomes by ICE race + income scores.

	Tertile 1 N(%)	Tertile 2 N(%)	Tertile 3 N(%)	*P* value
Infant <7 day mortality	94 (31.54)	109 (36.58)	95 (31.88)	0.49
Infant mortality before discharge	142 (34.8)	146 (35.78)	120 (29.41)	0.23
Infant > /=2 acute care visits	**1290 (36.38)**	**1239 (34.94)**	**1017 (28.68)**	**<0.001**
Infant readmission	**905 (34.35)**	**910 (34.54)**	**820 (31.12)**	**0.03**
Infant mortality after discharge	**46 (40)**	**45 (39.13)**	**24 (20.87)**	**0.02**

Bold type indicates statistical significance *p* < 0.05.

Among all birthing people with preterm infants, Black birthing people in the least privileged ICE race + income categories, tertile 1 (RR 1.27, 95% CI 1.18–1.36) and tertile 2 (RR 1.21 95% CI 1.12–1.30) were more likely to have infants who were seen at 2 or more acute care visits (Table 4). These findings persisted when adjusting for infant characteristics and birthing person characteristics, but after adjusting for social factors in model 4 the findings were attenuated (RR 1.12 95% CI 0.96–1.29). Similarly, birthing people in the least privileged tertiles were more likely to have infants who were readmitted to the hospital (model 1 RR 1.10 95% CI 1.01–1.20). Following adjustment for infant characteristics, point estimates were unchanged. When additionally adjusting for birthing person characteristics the least privileged group, tertile 1, continued to have increased risk for readmission (model 3 RR 1.21 95% CI 1.02–1.43) but no differences were found when additionally adjusting for social factors (model 4 RR 1.15 95% CI 0.96–1.38). In tertile 2, findings were attenuated when additionally adjusting for birthing person characteristics (model 3 1.11 95% CI 0.94–1.31).

**Table 4:** Risk ratios of ICE score tertiles and adverse postnatal outcomes for <37 week infants.

	Model 1	Model 2	Model 3	Model 4
Infant <7 day mortality				
Tertile 1	0.99 (0.75, 1.30)	0.97 (0.74, 1.29)	0.95 (0.55, 1.65)	1.01 (0.55, 1.83)
Tertile 2	1.14 (0.87, 1.50)	1.14 (0.87, 1.50)	1.35 (0.80, 2.26)	1.43 (0.82, 2.47)
Tertile 3	Reference	Reference	Reference	Reference
Infant before discharge mortality				
Tertile 1	1.17 (0.93, 1.48)	1.16 (0.92, 1.47)	1.09 (0.69, 1.73)	1.29 (0.78, 2.13)
Tertile 2	1.21 (0.96, 1.53)	1.21 (0.96, 1.54)	1.30 (0.84, 2.02)	1.46 (0.90, 2.37)
Tertile 3	Reference	Reference	Reference	Reference
Infant > /=2 acute care visits				
Tertile 1	**1.27 (1.18, 1.36)**	**1.27 (1.18, 1.36)**	**1.24 (1.08, 1.42)**	1.12 (0.96, 1.29)
Tertile 2	**1.21 (1.12, 1.30)**	**1.21 (1.12, 1.30)**	**1.18 (1.02, 1.35)**	1.11 (0.96, 1.28)
Tertile 3	Reference	Reference	Reference	Reference
Infant readmission				
Tertile 1	**1.10 (1.01, 1.20)**	**1.09 (1.00, 1.19)**	**1.21 (1.02, 1.43)**	1.15 (0.96, 1.38)
Tertile 2	**1.11 (1.02, 1.20)**	**1.10 (1.01, 1.20)**	1.11 (0.94, 1.31)	1.09 (0.91, 1.31)
Tertile 3	Reference	Reference	Reference	Reference
Infant after discharge mortality				
Tertile 1	**1.92 (1.17, 3.14)**	**1.91 (1.16, 3.11)**	**4.09 (1.15, 14.5)**	^a^
Tertile 2	**1.88 (1.14, 3.08)**	**1.88 (1.14, 3.08)**	2.67 (0.70, 10.1)	^a^
Tertile 3	Reference	Reference	Reference	Reference

Bold typeface indicates statistical significance *p* < 0.05.

Model 1: Unadjusted.

Model 2: Adjusted for gestational age, growth (SGA, LGA), sex.

Model 3: Additionally adjusted for birthing person medical factors: age, BMI, smoking, drug/alcohol use, hypertension, diabetes, and prenatal care.

Model 4: Additionally adjusted for birthing person education, insurance status, and WIC use.

^a^Sample size too small to analyze.

Black birthing people living in the least privileged areas (tertile 1 and 2) were more likely to have infants who died after hospital discharge (model 1 RR 1.92 95% CI 1.17–3.14, RR 1.88 95% CI 1.14–3.08). These findings persisted after adjusting for infant characteristics (model 2 RR 1.91 95% CI 1.16–3.11, RR 1.88 95% CI 1.14–3.08). When adjusting for birthing person characteristics the least privileged tertile continued to have a higher risk of infant mortality after discharge and tertile 2 was not different from the referent group (model 3 RR 4.09 95% CI 1.15–14.5, RR 2.67 95% CI 0.7–10.1). The sample size for model 4 was too small to analyze.

No difference in ICE metrics were found between the least privileged group (tertile 1) and the most privileged group for <7 day mortality or before discharge mortality (model 1 RR 0.99 95% CI 0.75–1.30, RR 1.17 95% CI 0.93–1.48). Results did not significantly differ after adjusting for infant, birthing person, or social factors.

## Discussion

In this study, we found that structural racism, as measured by racial and economic segregation via the Index of Concentration at the Extremes (ICE), was associated with poor postnatal outcomes for Black preterm infants. Black birthing people who delivered preterm and lived in less privileged areas were consistently at higher risk for frequent infant acute care visits, rehospitalizations, and death after hospital discharge compared to those who lived in more privileged areas.

Our study is consistent with both historical and scientific literature describing the negative impact of structural racism and segregation to the health and wellbeing of Black communities.^28^ Historically, the U.S. has enacted deliberately and overtly discriminatory laws and practices, particularly transparently in the Jim Crow era, that have resulted in disparate access to social determinants of health (SDH) including but not limited to quality education, housing, employment, and wealth.^10,29–31^ Although enslavement and legalized discrimination have been abolished, the legacy of structural racism continues. In medical literature, previous studies have identified structural racism as a significant contributor to racial disparities in perinatal outcomes. Redlining, an example of a structurally racist policy, and the resultant segregation in the U.S. have been associated with poor perinatal outcomes, including preterm birth, low birthweight, low Apgar scores, increased likelihood of NICU admission, and preterm comorbidities like intraventricular hemorrhage.^13,17,32–37^ Structural racism as measured by ICE has been associated with perinatal outcomes including PTB and IMR.^15^ Fewer studies link structural racism and postnatal outcomes for preterm infants; we identified one study describing an association between neighborhood inequality and emergency department utilization for NICU graduates.^22^ Another study described an association between ICE and a combined death and severe preterm comorbidity (necrotizing enterocolitis, intraventricular hemorrhage, retinopathy of prematurity, and bronchopulmonary dysplasia) outcome.^35^ To our knowledge, ICE as a measure of structural racism has not been associated with infant postnatal healthcare utilization or infant mortality relative to discharge. Our study is consistent with previous studies and extends our understanding of the impact of structural racism beyond the hospital for infants who are born preterm. Additionally, it provides reinforcement to the concept of structural racism contributing to poor postnatal outcomes for preterm infants. Identifying structural racism as a primary cause of racial disparities in the postnatal period is the first step to prioritizing and implementing appropriate structural interventions to improve outcomes.

Proposed pathophysiologic mechanisms and causal pathways linking segregation to poor perinatal outcomes include lower-quality care, stress exposure, socioeconomic disadvantage, and environmental toxins such as exposure to air pollution and lead.^38–42^ Environmental factors are known to be associated with preterm birth and disproportionately burden Black communities. Similar prenatal and postnatal environmental and stress exposures may contribute to poor postnatal outcomes but we did not have relevant data to test this hypothesis in this study. Adult medical conditions, similar to birthing people medical conditions included in the study, are also impacted by structural racism.^18–21^ Thus, covariates included may operate on the causal pathway from structural racism to adverse postnatal outcomes.

Notably, adjusting for infant and birthing person characteristics did not significantly change the likelihood of adverse postnatal outcomes between groups. However, adjusting for SDH including insurance status, education, and participation in a federal income supplementation program, attenuated the risk in our last model, suggesting that the SDH chosen were important mediators of structural racism and poor postnatal outcomes. This is consistent with our framework of structural racism operating through the unequal distribution of SDH to impact postnatal outcomes. Our findings suggest that racism continues to negatively impact Black birthing people and their infants.^34^ We cannot exclude the possibility of collinearity between these social determinants given their relationships with income. We did not find an association between structural racism and pre-discharge or less than 7-day mortality. Previous studies have found associations between structural racism and infant mortality in the first year of life, but we are not aware of any studies that examine associations between structural racism and less than 7 day mortality or mortality relative to discharge.^15–17,22–24^ Previous studies have not shown racial disparities for in-hospital infant mortality, thus it is not surprising we did not find an association with structural racism for this outcome.^1,4,43^

As ICE only uses race and income, it measures a small component of structural racism and therefore does not fully capture the impact of structural racism and lived experience of Black individuals in the U.S. Our study was limited by the variables in our datasets and important SDH data regarding employment, housing, wealth, and environmental exposures were not available to us. Similarly, self-reports of racism and discrimination to understand the lived-experiences of Black birthing people are not included in these census and administrative datasets. The demographics and politics of California do not reflect the demographics and politics of the nation, thus study results may not be directly generalizable to the entire country. Although structural racism is pervasive across geographic regions and populations in the U.S., the extent to which groups have experienced local, state, and federal support varies. For example, since study completion, the COVID-19 pandemic has highlighted exacerbated racial income and health inequities and variable responses by local, state, and federal governments. Our study is limited to the pre-pandemic era, and thus cannot be fully generalizable to pandemic or post-pandemic eras, but worsening societal inequities are worrisome for impact on preterm infant outcomes. Strengths of this study include using large, diverse population datasets and a frequently used measure of structural racism to examine previously difficult to study and rare outcomes.^15,19–22,44,45^ Other populations that are historically marginalized, including Hispanic and Indigenous populations, have also experienced structural racism. Further investigation is needed to examine the impact of structural racism on preterm infant outcomes in these populations.

Structural racism is a fundamentally systemic problem that can only be solved through fundamentally systemic solutions by those with the power to decide, the power to act, and the control over the resources.^46^ Structural racism is the result of disparate laws and practices that have resulted in disparate resources and opportunities for racialized groups, such as income and wealth inequality.^10^ Systemic societal change to promote equity across federal, institutional, and interpersonal levels, has the potential to improve health outcomes when all birthing persons and their children can live up to their highest potential.^46^ For example, addressing structural racism through federal policies can include providing opportunities for basic income, housing, healthcare, and employment for historically marginalized groups. Medical institutions can evaluate and address current culture and practice differences, strengthen ties to community resources, and reduce additional financial burdens.^38,47^ Both the federal government and medical institutions must both examine how structural racism is operationalized within their spaces and redistribute and equitably share power for all by revising their laws, practices, policies, and culture to become actively antiracist.^47–49^

## Conclusion

In our study, structural racism, measured by racial and economic segregation, was associated with adverse postnatal outcomes for preterm infants born to Black birthing people, including frequent acute care visits, readmissions, and post-discharge mortality. Future studies and interventions that prioritize dismantling structural racism have the potential to achieve equitable outcomes for preterm infants.

## Data Availability

The datasets analyzed during the current study are available from the corresponding author on reasonable request.
